# Mesenchymal Stem Cells Restore Endometrial Integrity and Sustain Pregnancy via CCR7‐ERK/JNK Signaling Modulation

**DOI:** 10.1155/sci/9963200

**Published:** 2026-01-16

**Authors:** Yunguo Lei, Juanmei Gao, Ning Zhang, Yunjun Lin, Weihua Yang, Yuequn Chen, Qiang Wei, Jianmei Xia

**Affiliations:** ^1^ Department of Gynecology and Obstetrics, Affiliated Hangzhou First People’s Hospital, School of Medicine, Westlake University, Hangzhou, 310006, China, westlake.edu.cn; ^2^ Zhejiang University School of Medicine, Hangzhou, 310003, China, zju.edu.cn; ^3^ Department of Hepatobiliary and Pancreatic Surgery and Minimally Invasive Surgery, Zhejiang Provincial People’s Hospital, Affiliated People’s Hospital, Hangzhou Medical College, Hangzhou, 310014, China, hznu.edu.cn

**Keywords:** CCR7, MAPK signaling pathways, mesenchymal stem cells (MSCs), NK cells, recurrent miscarriage (RPL)

## Abstract

Recurrent pregnancy loss (RPL), defined as more than two consecutive miscarriages before 20 weeks of gestation, affects 1%–5% of reproductive‐aged women, with nearly half of the cases remaining idiopathic. Using ethanol‐induced endometrial injury in rats to simulate RPL, we demonstrated that mesenchymal stem cells (MSCs) exert therapeutic effects through two complementary mechanisms: CCR7‐mediated endometrial repair and immunomodulation of uterine natural killer (uNK) cells, compared to ethanol‐induced injury, this treatment achieved a 190% increase in embryo retention (from 2.4 to 7 embryos). Transcriptomic analysis and Western blotting of MSC‐cocultured NK92 cells revealed significant CCR7 upregulation (1.75‐fold increase at the optimal dose of 2 × 10^4^ cells/mL MSCs) and activation of NK differentiation pathways. This was corroborated by immunofluorescence showing enhanced CCR7^+^ NK cell infiltration in MSC‐treated endometria. MSCs administration altered cytokine profiles by decreasing pro‐inflammatory mediators (IL‐6, TNF‐α, and IL‐1β) and increasing anti‐inflammatory IL‐10 levels simultaneously. Mechanistically, MSCs orchestrate endometrial repair through sequential events: induce the formation of a gradient of CCR7 expression on the endometrial layer and the surface of NK cells; followed by ERK/JNK pathway activation, which promotes CCR7^+^ uNK cell generation; and finally initiates endometrial proliferation with an increased proportion of Ki67^+^ cells. Our integrated multi‐omics approach—combining RNA‐Seq, protein analysis, and cytokine profiling—establishes the CCR7‐ERK/JNK axis as a promising therapeutic target, providing clinically relevant parameters for MSCs dosing and administration protocols in idiopathic RPL management.

## 1. Introduction

Recurrent pregnancy loss (RPL), defined as two or more consecutive spontaneous miscarriages before 20 weeks of gestation, affects 1%–5% of reproductive‐aged women and poses substantial physical and psychological burdens [1–3]. While chromosomal abnormalities and thrombophilia account for ~50% of cases, nearly half of RPL diagnoses remain idiopathic, underscoring the urgent need to elucidate novel pathophysiological mechanisms [4]. Emerging evidence suggests that dysregulated uterine immune homeostasis is a pivotal contributor, particularly impaired natural killer (NK) cell dynamics at the fetal–maternal interface [5, 6]. Uterine NK (uNK) cells, a specialized CD56^+^CD16^-^ subset, orchestrate critical processes including spiral artery remodeling, trophoblast invasion, and endometrial stromal repair through cytokine/chemokine secretion and direct cellular interactions [7, 8]. Aberrant uNK cytotoxicity or defective migration has been strongly associated with disrupted decidualization and pregnancy failure in clinical cohorts [9].

Mesenchymal stem/stromal cells (MSCs) have emerged as potent immunomodulators capable of restoring tissue homeostasis in reproductive pathologies [10]; they can repair damaged endometrium, regulate immune imbalance, and delay aging, thereby improving pregnancy rates and outcomes. Notably, recent studies revealed that MSCs transplantation significantly reduces peripheral NK cytotoxicity while promoting uterine CD49a^+^ tissue‐resident NK cell accumulation in unexplained RPL patients [11]. Paradoxically, while MSCs are known to inhibit NK cell‐mediated alloreactivity in transplantation settings, their capacity to enhance NK cell recruitment and tissue‐specific functional reprograming in pregnancy maintenance remains mechanistically undefined.

A key unresolved question centers on how MSCs spatially coordinate NK cell trafficking and activation within the endometrium. The chemokine receptor CCR7, traditionally recognized for directing lymphocyte homing to lymphoid organs, has recently been implicated in uNK cell migration during early gestation [12, 13].Intriguingly, CCR7 ligands (CCL19/21) are abundantly expressed in decidual stromal cells, suggesting a potential crosstalk axis between MSCs‐modulated stromal niches and NK cell dynamics [14]. Furthermore, downstream ERK/JNK signaling pathways, critical regulators of NK cell chemotaxis and cytotoxic granule polarization, may serve as convergent effectors of CCR7‐mediated functional reprograming [15–17]. Yet, the triad relationship linking MSCs paracrine signaling, CCR7‐dependent NK cell trafficking, and endometrial stromal repair has never been systematically investigated.

In this study, we hypothesize that MSCs prevent pregnancy loss through a dual mechanism: (1) restoring endometrial stromal homeostasis through CCR7‐mediated ERK/JNK activation, and (2) reprograming uNK cell migration/activation dynamics to maintain fetomaternal tolerance. By using a well‐characterized rat RPL model, combined with transcriptomics, we demonstrate that in utero MSCs transplantation activates CCR7‐ERK /JNK signaling, driving uNK cell recruitment and a functional transition from cytotoxic to promoting endometrial repair. These findings establish a previously unrecognized mesenchymal‐immune axis critical for pregnancy maintenance, providing a mechanistic basis for MSCs‐based therapies for RPL.

## 2. Materials and Methods

### 2.1. Animals and RPL Model Establishment

Female Sprague–Dawley rats (*n* = 40; 8–10 weeks old; 220 ± 15 g body weight) were purchased from Shanghai Model Organisms Center, Inc. After 1 week of acclimatization under specific pathogen‐free conditions (temperature: 22 ± 1°C, humidity: 55 ± 5%, 12 h light/dark cycle), rats were randomly assigned into four experimental groups. (1) PBS control (*n* = 10): Intrauterine infusion of 100μL phosphate‐buffered saline (HyClone); (2) ethanol‐induced RPL model (*n* = 10): Intrauterine injection of 95% ethanol (Sigma–Aldrich) (100μL) to induce endometrial injury and miscarriage; (3) low‐dose MSCs treatment (*n* = 10): Ethanol injury + 1 × 10^5^MSCs via intrauterine infusion; (4) high‐dose MSCs treatment (*n* = 10): ethanol injury + 4 × 10^5^MSCs (same administration route and timing). All animal procedures were approved by the Institutional Animal Care and Use Committee under the ethics number IACUC‐20221123‐03. MSCs were given in utero on the same day as alcohol treatment. On Day 21 after the embryo transfer, we collected samples to count embryos and analyze serum and uterine tissue.

### 2.2. Umbilical Cords MSCs Isolation

MSCs were isolated from the umbilical cords of healthy parturients who underwent elective cesarean section in the Department of Obstetrics, Hangzhou First People’s Hospital. All participants provided written informed consent, and the study protocol was reviewed and approved by the Ethics Committee of Hangzhou First People’s Hospital (Approval Number: ZN‐20211227‐0147‐01). Inclusion criteria for the healthy pregnant women included full‐term gestation (37–40 weeks), absence of pregnancy complications such as preeclampsia, gestational diabetes, or infections, and no history of obstetric disorders.

Umbilical cords were collected immediately after delivery, with care taken to avoid blood contamination. After transportation to the laboratory, the umbilical cords were carefully washed with PBS to remove any remaining blood and debris. The tissues were then processed by cutting them into small segments (~1 cm in length). The small pieces of tissue were placed in sterile culture plates and digested using an enzymatic solution containing 0.1% collagenase type I (Sigma–Aldrich, USA) and 0.1% hyaluronate (Sigma–Aldrich, USA). The digestion process was performed at 37°C for 1–2 h with gentle agitation to facilitate cell dissociation.

After digestion, the cell suspension was filtered through a 70 µm cell strainer (Thermo Fisher Scientific, USA) to remove tissue debris, and the collected cells were centrifuged at 300 × *g* for 10 min. The pelleted cells were resuspended in complete medium, which consisted of Dulbecco’s Modified Eagle Medium (DMEM) supplemented with 10% fetal bovine serum (FBS) and 1% penicillin–streptomycin (Gibco, USA). The cells were plated at a density of 1 × 10^4^ cells/cm^2^ and cultured in a humidified incubator at 37°C with 5% CO_2_. The medium was replaced every 3–4 days, and nonadherent cells were removed. After 7–10 days, the adherent cells that exhibited the characteristic spindle‐shaped morphology of MSCs were expanded and used for further analysis.

### 2.3. Characterization of UCMSCs

The isolated MSCs were characterized for their phenotype by flow cytometry analysis. Cells were stained for surface markers CD44, CD73, and CD90 to confirm their mesenchymal lineage, and for MHC Ⅱ、CD34 and CD45 to ensure the absence of hematopoietic markers. The cells were also assessed for their ability to differentiate into osteocytes, adipocytes, and chondrocytes following standard differentiation protocols.

### 2.4. Coculture of NK92 Cells and MSCs

The human NK92 cell line was maintained in RPMI‐1640 medium (Gibco, 11875093) supplemented with 10% heat‐inactivated FBS (Gibco, 10,099,141), 1% penicillin–streptomycin (HyClone, SV30010), and recombinant human IL‐2 (500 U/mL; PeproTech, 200) at 37°C under 5% CO_2_. To investigate MSCs‐mediated immunomodulation, a dual‐chamber transwell system (Corning, 0.4 μm polyester membrane, CLS3470) was employed under serum‐free conditions (X‐VIVO15, Monza, BE02‐060 F). Experimental setup: upper chamber: 1 × 10^4^, 2 × 10^4^, and 4 × 10^4^ P3 MSCs in 200 μL basal medium, respectively; lower chamber: 5 × 10^5^ NK92 cells in 800 μL complete medium (IL‐2 free); control: NK92 monoculture with equivalent IL‐2 deprivation. Following 48‐h coculture, NK92 cells were harvested via gentle pipetting.

### 2.5. CCR7 Knockdown in NK‐92 Cells

NK92 cells were maintained in RPMI‐1640 medium supplemented with 10% FBS, 500 U/mL IL‐2, and 1% penicillin–streptomycin at 37°C in 5% CO_2_. For siR‐CCR7 knockdown, cells were transfected with 50 nM siRCCR7 (or scrambled control siRNA) using Lipofectamine RNAiMAX (Thermo Fisher) according to the manufacturer’s protocol. Transfection efficiency was validated by qPCR (48 h posttransfection) using primers targeting CCR7 (forward: CAACATCACCAGTAGCACCTGTG; reverse: TGCGGAACTTGACGCCGATGAA).

### 2.6. RNA‐Seq Analysis

NK92 cells (monoculture vs. MSCs coculture underwent RNA extraction using TRIzol, with library preparation (NEBNext Ultra II) and Illumina NovaSeq 6000 sequencing (150 PE, 40M reads/sample). Gene ontology (GO) and Kyoto Encyclopedia of Genes and Genomes (KEGG) pathway enrichment analyses were performed to identify key molecular pathways involved in MSCs‐mediated regulation of NK cell function.

### 2.7. Serum Cytokine Analysis by ELISA

Blood samples were collected via abdominal aorta puncture from rats on gestational day (GD) 21 under anesthesia. Serum was separated by centrifugation (3000 × *g*, 15 min, 4°C) and stored at −80°C until analysis. Cytokine levels (IL‐6, IL‐10, IL‐1β, and TNF‐α) were quantified using commercial ELISA kits (Abclonal, Wuhan, China) according to the manufacturer’s instructions. Specific catalog numbers: IL‐6 (RK00004), IL‐10 (RK00050), IL‐1β (RK00009), and TNF‐α (RK00029). Briefly, 100 μL of serum or standard (IL‐6:15.6–1000 pg/mL; IL‐10:7.8–500 pg/mL; IL‐1β: 31.2–2000 pg/mL; TNF‐α: 62.5–4,000 pg/mL) was added to precoated 96‐well plates. After incubation (37°C, 2 h), wells were washed and incubated with biotinylated detection antibodies, followed by streptavidin‐HRP (30 min) and TMB substrate (15 min). Reactions were stopped with 2N H_2_SO_4_, and absorbance was measured at 450 nm (BioTek Synergy H1).Standard curves were generated using four‐parameter logistic regression in GraphPad Prism 9.0. All samples were tested in duplicate. Statistical differences between groups were assessed by one‐way ANOVA with Tukey’s post hoc test ( ^∗^ *p*  < 0.05,  ^∗∗^ *p*  < 0.01,  ^∗∗∗^ *p*  < 0.001).

### 2.8. Data Analysis

All experiments were conducted with ≥3 biological replicates demonstrating consistent reproducibility. Quantitative data are presented as mean ± SEM from independent experiments. Statistical comparisons were performed using GraphPad Prism (versions 5–8; v9.0 for ANOVA analyses) with the following approaches: pairwise comparisons: two‐tailed Student’s t‐tests (paired/unpaired as appropriate); multigroup comparisons: one‐way ANOVA with Dunnett’s post hoc test (vs. control group); Significance thresholds were defined as:  ^∗^ *p*  < 0.05,  ^∗∗^ *p*  < 0.01,  ^∗∗∗^ *p*  < 0.001 (ns = nonsignificant).

## 3. Results

### 3.1. Dose‐Dependent Ethanol‐Induced Endometrial Injury in a Rat Model

We investigated the therapeutic effects of MSCs on ethanol‐induced endometrial injury using a model in 12‐week‐old rats. The rats were treated with PBS, ethanol, or ethanol + MSCs, with tissues collected 4 weeks posttreatment for histological analysis (Figure 1A). Morphological and histological analyses revealed dose‐dependent ethanol‐induced endometrial injury, with maximal damage observed at 0.3–0.4 μL/g concentrations. Gross examination revealed significant uterine wall thinning in ethanol‐treated groups compared to PBS controls (Figure 1B). H&E staining confirmed structural disruption characterized reduction in glandular density and complete loss of epithelial stratification at high doses (Figure 1C), accompanied by prominent lymphocytic infiltration in the stroma. Masson’s trichrome staining showed decreased collagen deposition (0.3 μL/g vs. PBS, *p*  < 0.05) and disrupted tissue architecture featuring functional layer atrophy (Figure 1D, G). Quantitative analysis confirmed (1) endometrial thickness reduction (*p*  < 0.05; Figure 1E) (2) dose‐dependent gland count decrease (*p*  < 0.05; Figure 1F); establishing 0.3 μ L/g as the threshold for significant histological damage.

Figure 1Dose‐dependent ethanol‐induced endometrial injury in a rat model. (A) Schematic illustration of ethanol‐induced endometrial injury modeling. (B) Macroscopic uterine morphology following graded ethanol treatments (0.2, 0.3, 0.4 μL/g). (C) Representative H&E‐stained endometrial sections showing ethanol dose‐dependent structural damage (scale bar:500 μm). (D) Masson’s trichrome staining demonstrating collagen deposition patterns (scale bar:500 μm). (E) Quantitative analysis of endometrial thickness. (F) Glandular count per high‐power field. (G) Collagen area quantification. Data expressed as mean ± SD (*n* = 4/group, “ns” stands for *p* > 0.05,  ^∗^ *p* < 0.05,  ^∗∗^ *p* < 0.01,  ^∗∗∗^ *p* < 0.001,  ^∗∗∗∗^ *p* < 0.0001).

**Figure:**
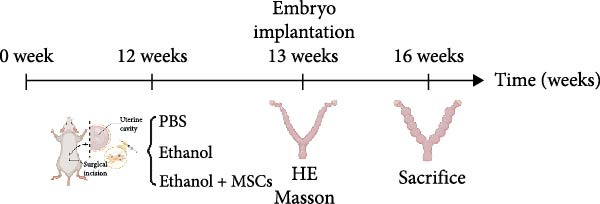
(A)

**Figure:**
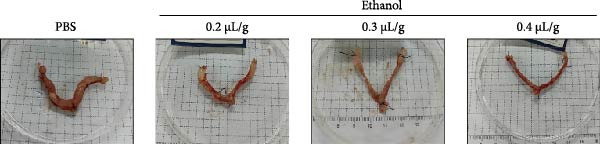
(B)

**Figure:**
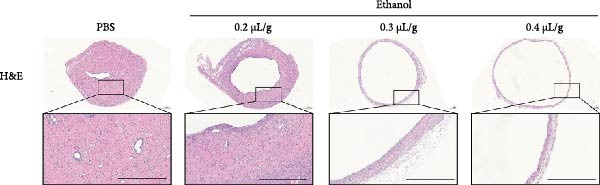
(C)

**Figure:**
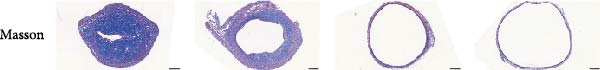
(D)

**Figure:**
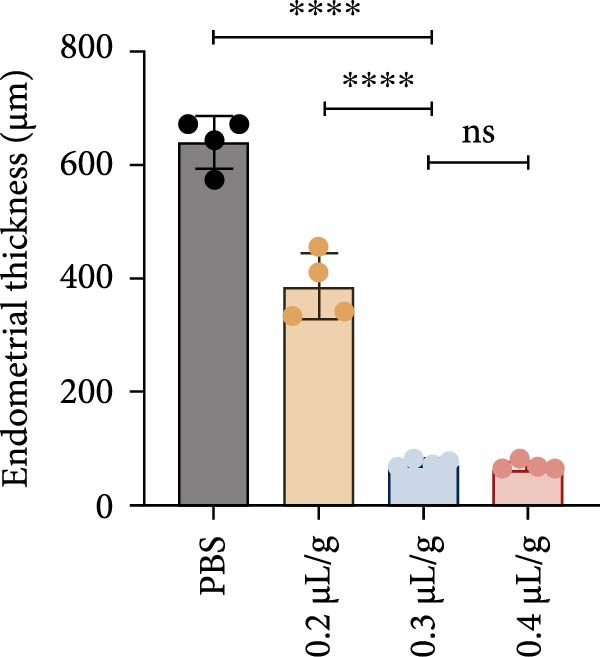
(E)

**Figure:**
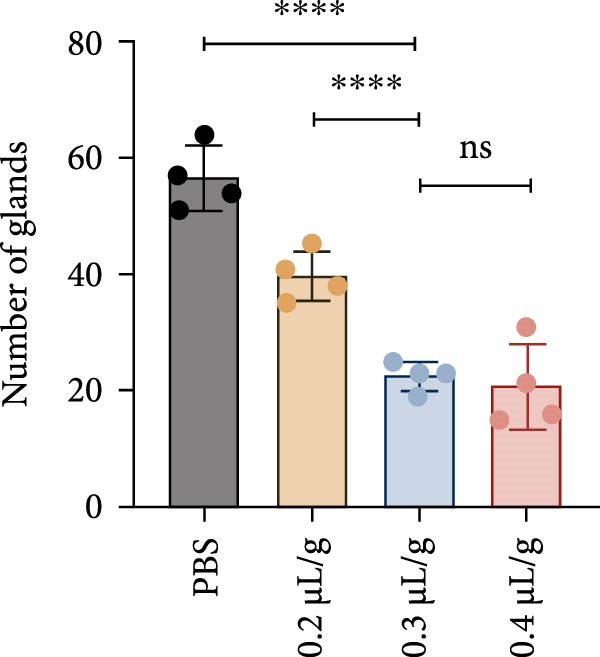
(F)

**Figure:**
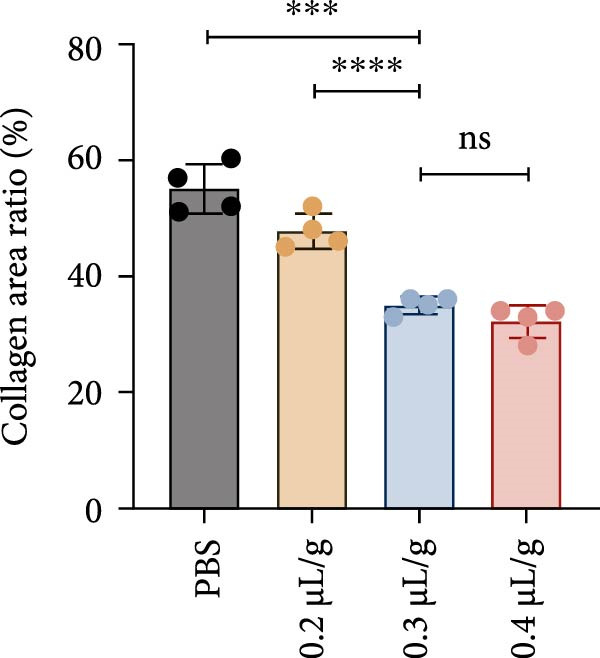
(G)

### 3.2. Differentiation and Characterization of MSCs

Bright‐field optical microscope revealed that both P0 and P1 generation umbilical cord‐derived MSCs exhibited characteristic spindle‐shaped, fibroblast‐like morphology typical of mesenchymal lineage cells (Figure 2A, B). In vitro trilineage differentiation assays confirmed their multipotency (1) osteogenic differentiation after 21 days demonstrated robust calcium deposition (Alizarin Red S^+^, Figure 2C) (2) adipogenic induction for 14 days yielded intracellular lipid droplet accumulation (Oil Red O^+^, Figure 2D). Flow cytometry analysis indicated >95% of cells expressed canonical MSCs markers (CD44^+^/CD73^+^/CD90^+^) while lacking hematopoietic (CD34^-^/CD45^-^) and MHC class II (HLA‐DR‐) markers (Figure 2E). Importantly, tail vein‐administered MSCs showed no tumorigenicity in nude mice at 2 weeks postinjection, in contrast to PC3 cell controls, which formed detectable tumors under identical conditions (Figure 2F), confirming their safety profile for therapeutic applications.

Figure 2Differentiation and characterization of MSCs. (A) Primary UC‐MSCs (P0) exhibiting typical fibroblast‐like morphology (scale bar:100 μm). (B) P1 UC‐MSCs displaying homogeneous spindle‐shaped morphology (scale bar:100 μm). (C) Osteogenic differentiation confirmed by Alizarin Red S staining (scale bar:100 μm). (D) Adipogenic differentiation verified by Oil Red O staining (scale bar:100 μm). (E) Flow cytometry analysis demonstrating CD90^+^/CD44^+^/CD73^+^/CD34^−^/CD45^−^/MHCⅡ^−^ phenotype. (F) Biosafety of MSCs demonstrated by comparing PC3 prostate cancer cells 2 weeks after tail vein injection in nude mice.

**Figure:**
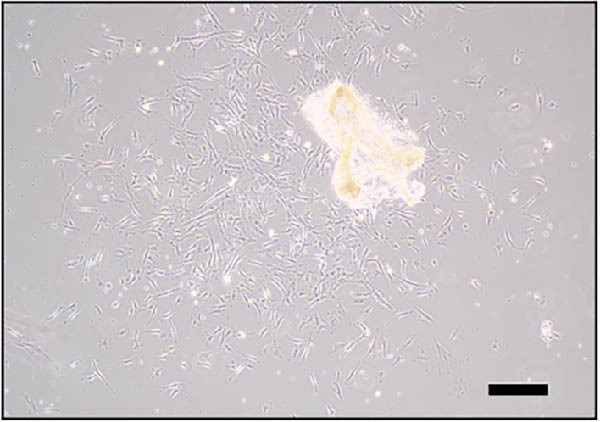
(A)

**Figure:**
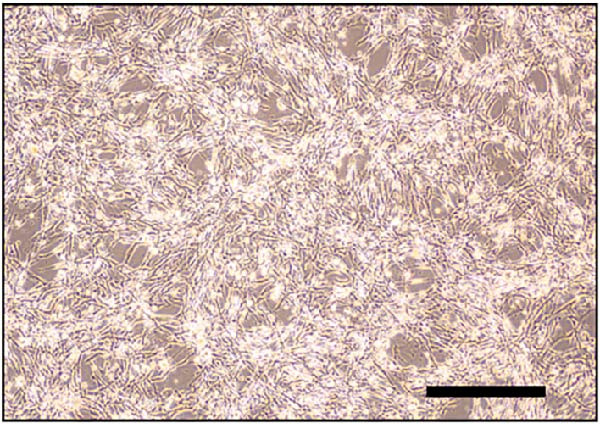
(B)

**Figure:**
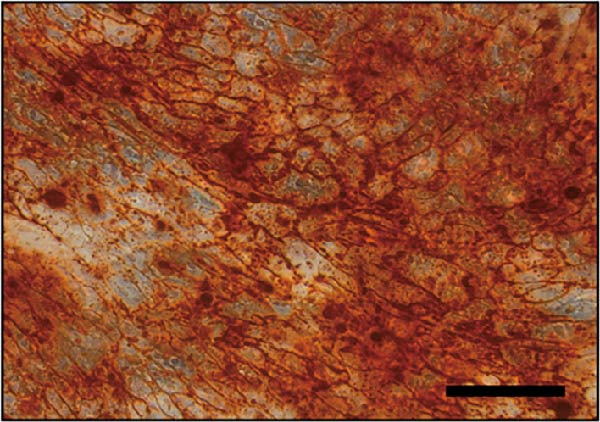
(C)

**Figure:**
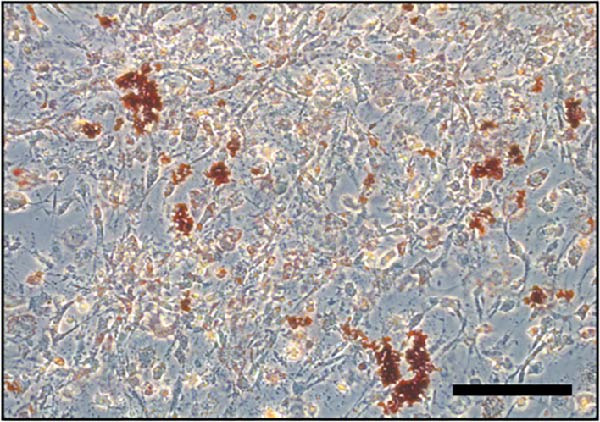
(D)

**Figure:**
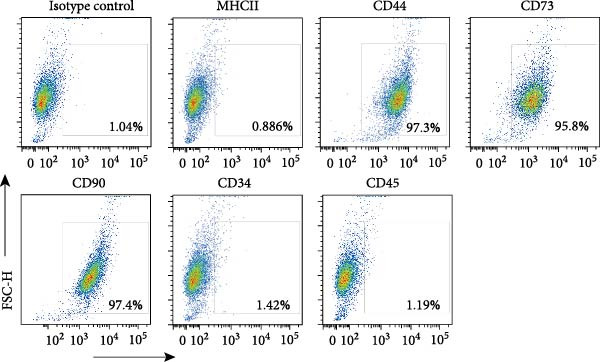
(E)

**Figure:**
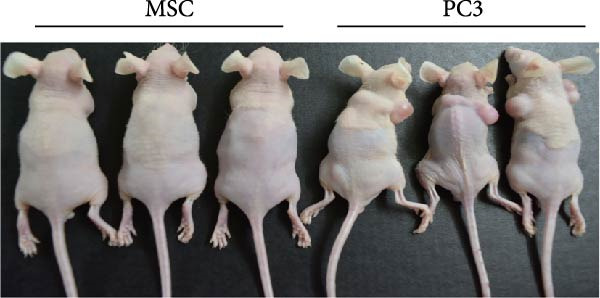
(F)

### 3.3. RNA‐Seq Analysis Reveals Key Pathways in NK92 Cells Following MSCs Coculture

To investigate the immunomodulatory effects of MSCs on NK cells—a key regulator of endometrial homeostasis, we established a coculture system and performed comparative transcriptomic analysis across three experimental groups: high‐dose MSCs vs. low‐dose MSCs, high‐dose MSCs vs. PBS, and low‐dose MSCs vs. PBS. Differential gene expression (DEG) analysis revealed extensive transcriptional reprograming, with the most pronounced changes observed in high‐dose MSCs vs. PBS (2876 upregulated and 2584 downregulated transcripts), followed by high‐dose vs. low‐dose MSCs (2546 up/2369 down) and low‐dose MSCs vs. PBS (2523 up/2230 down) (Figure 3A). Hierarchical clustering of the top 100 DEGs demonstrated group‐specific expression patterns, with CCR7 consistently upregulated in MSCs‐treated groups compared to PBS controls, implicating its potential role in MSCs‐NK crosstalk (Figure 3B). GO enrichment analysis of low‐dose MSCs vs. PBS highlighted significant terms (Top20 by *p*‐value), including regulation of NK cell differentiation (GO:0032826, *p*  < 0.05), suggesting MSCs treatment profoundly modulates NK cell functionality (Figure 3C). Further analysis of NK cell DEGs revealed enrichment in immune activation (GO:0002253, *p*  < 0.05), stress‐responsive MAPK signaling (GO:0032874, *p*  < 0.05), and inflammatory/metabolic pathways (GO:0002437, *p*  < 0.05), mechanistically supporting the CCR7‐ERK/JNK axis in NK cell adaptation and stromal repair (Figure 3D).

Figure 3RNA‐Seq analysis reveals key pathways in NK92 cells following MSCs coculture. (A) RNA‐Seq analysis reveals dose‐dependent transcriptional reprograming in NK cells. (B) Heatmap of differentially expressed genes (FDR < 0.05) highlights CCR7 as a key regulator. (C) Top 20 enriched GO terms in the low‐dose MSCs group vs. the PBS group (biological process). (D) Molecular function enrichment in the high‐dose MSCs group vs. the PBS group. Color scale: blue (low) to red (high expression).

**Figure:**
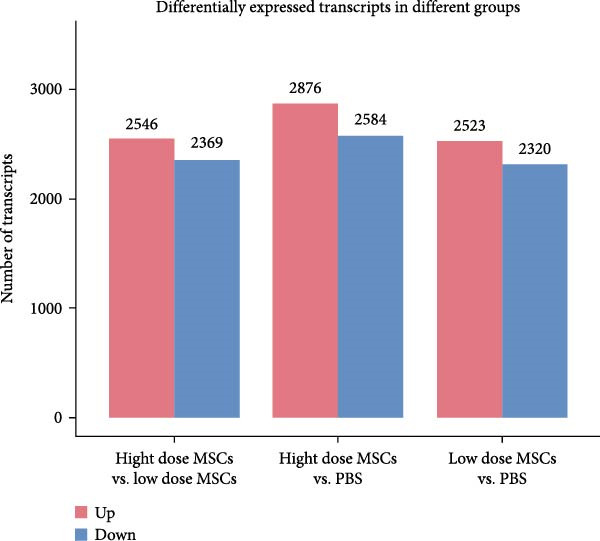
(A)

**Figure:**
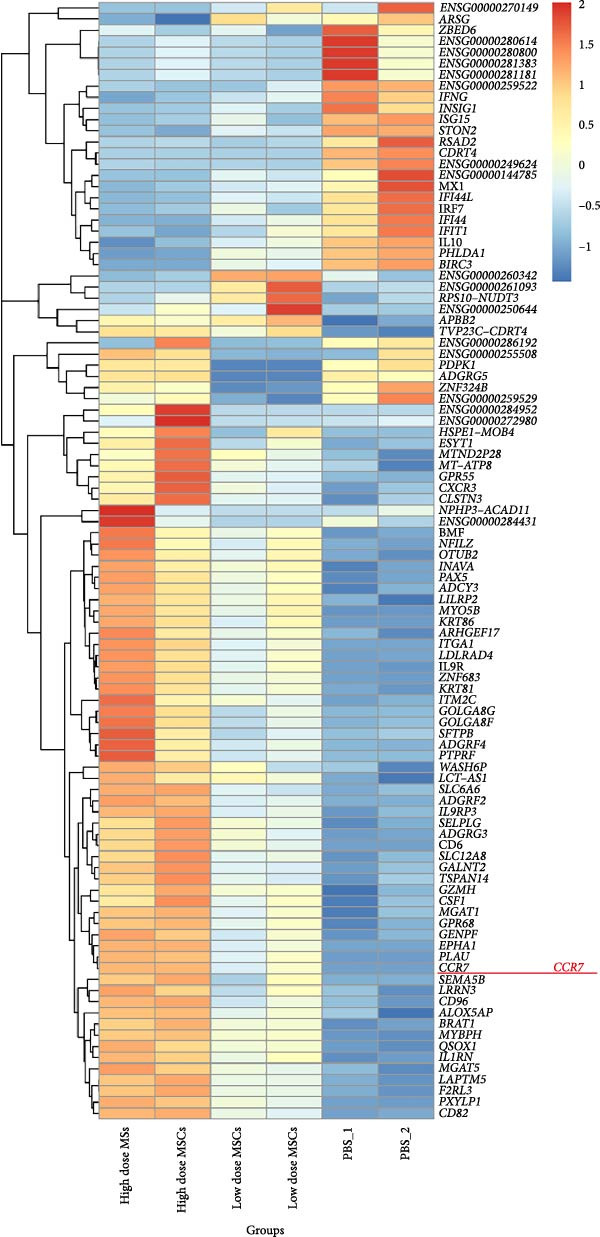
(B)

**Figure:**
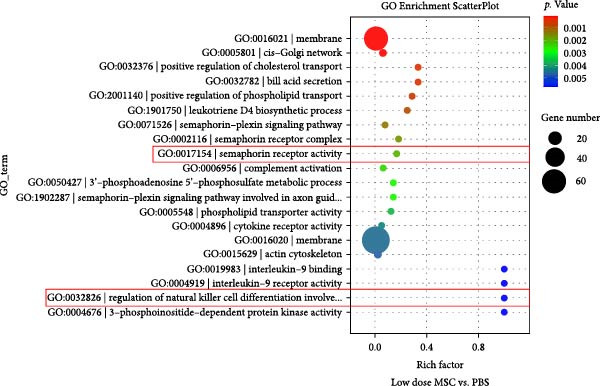
(C)

**Figure:**
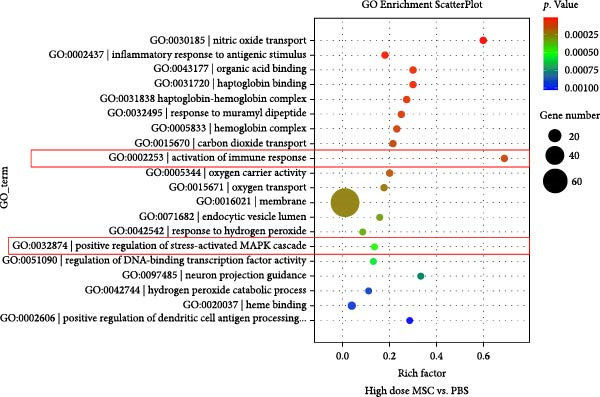
(D)

### 3.4. MSCs‐Mediated Endometrial Regeneration Associates With CCR7^+^NK Cell Infiltration in Ethanol Injury

Our study systematically investigated the protective effects of MSCs on ethanol‐damaged endometrium. Histological analysis (H&E and Masson staining, Figure 4 A) revealed that ethanol exposure led to endometrial atrophy, while MSCs treatment (low‐ and high‐dose groups) restored endometrial morphology and increased collagen accumulation (Figure 4D). Quantitative assessment further demonstrated that ethanol significantly decreased endometrial thickness (Figure 4B) and gland numbers (Figure 4C), whereas MSCs reversed these changes in a dose‐dependent manner. Embryo retention analysis revealed striking intergroup differences. The PBS control group maintained maximal embryonic viability, whereas ethanol exposure caused severe fetal loss. In contrast, the treatment group achieved a 190% increase in embryo retention (from 2.4 to 7 embryos) compared to the ethanol‐induced injury group. MSCs administration partially rescued embryonic viability in a dose‐dependent manner (*p*  < 0.001 vs. ethanol, Figure 4E). Immunofluorescence staining and Ki67 staining showed that ethanol suppressed endometrial cell proliferation and decreased the proportion of NCR^+^CCR7^+^NK cells, and MSCs restored cell proliferation and immune balance (Figure 4F). Serum ELISA analysis (Figure 4G) demonstrated that ethanol administration significantly elevated pro‐inflammatory cytokines (IL‐6, TNF‐α, and IL‐1β) while suppressing anti‐inflammatory IL‐10 levels, whereas MSCs treatment restored cytokine homeostasis toward physiological baseline (*p*  < 0.01 vs. ethanol group).Collectively, these findings suggest that MSCs exert protective effects on ethanol‐damaged endometrium by modulating inflammation and restoring endometrial structure, highlighting their potential for endometrial repair in clinical settings.

Figure 4MSCs‐mediated endometrial regeneration associates with CCR7^+^NK cell infiltration in ethanol injury. (A) Representative H&E and masson staining reveal structural recovery of endometrial glands, stroma, and collagen deposition after MSCs treatment. (B–E) Statistical analysis of embryo numbers, endometrial thickness, gland count, and collagen area in each group (*n* = 5/group,  ^∗^ *p* < 0.05,  ^∗∗^ *p* < 0.01,  ^∗∗∗^ *p* < 0.001,  ^∗∗∗∗^ *p* < 0.0001, scale bars:200 μm). (F) Representative immunofluorescence staining (upper and middle panels) and Ki67 immunohistochemistry (lower panels) of tissue sections from vehicle, ethanol, low‐dose MSCs, and high‐dose MSCs groups. Immunofluorescence images show costaining of DAPI (blue, nuclei), NCR (red, natural cytotoxicity receptor), and CCR7 (green). White boxes in the upper panels indicate regions magnified in the middle panels (IF, scale bars:20 μm; IHC, scale bars:50 μm). (G) Serum cytokine profiles demonstrating MSCs‐mediated IL‐6/TNF‐α /IL‐1 β reduction and IL‐10 elevation. Data: mean ± SEM (n = 5/group, *p* < 0.01 vs. ethanol group).

**Figure:**
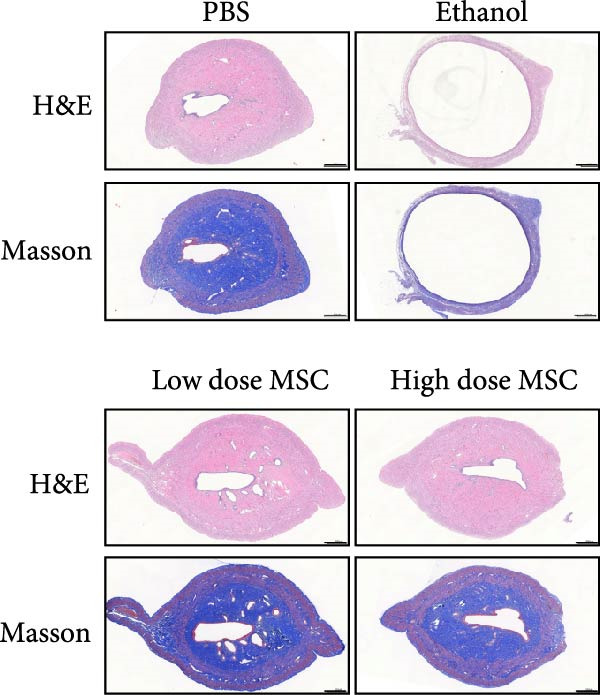
(A)

**Figure:**
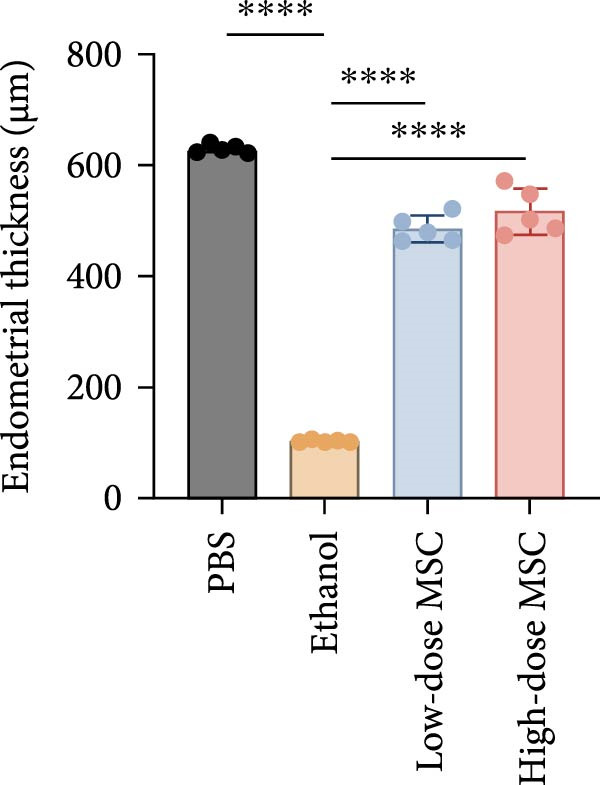
(B)

**Figure:**
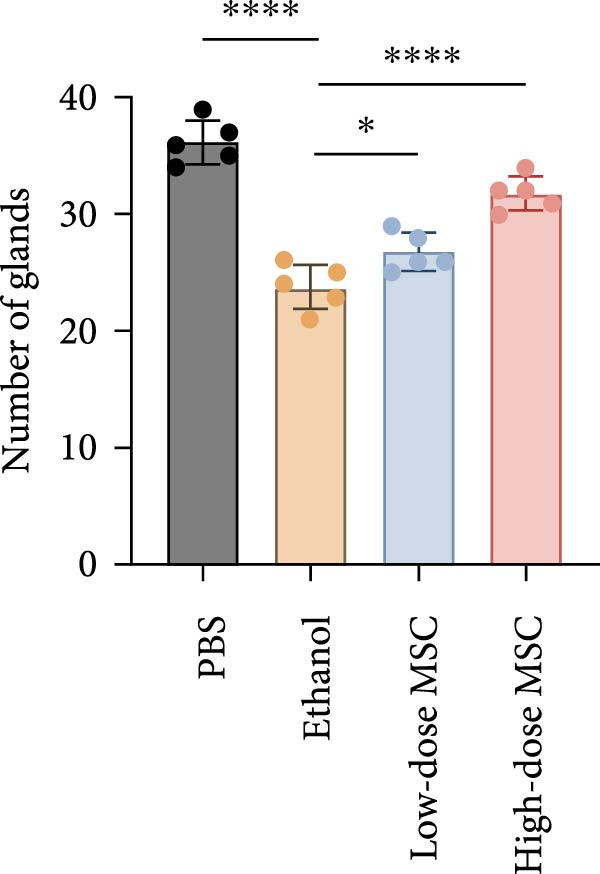
(C)

**Figure:**
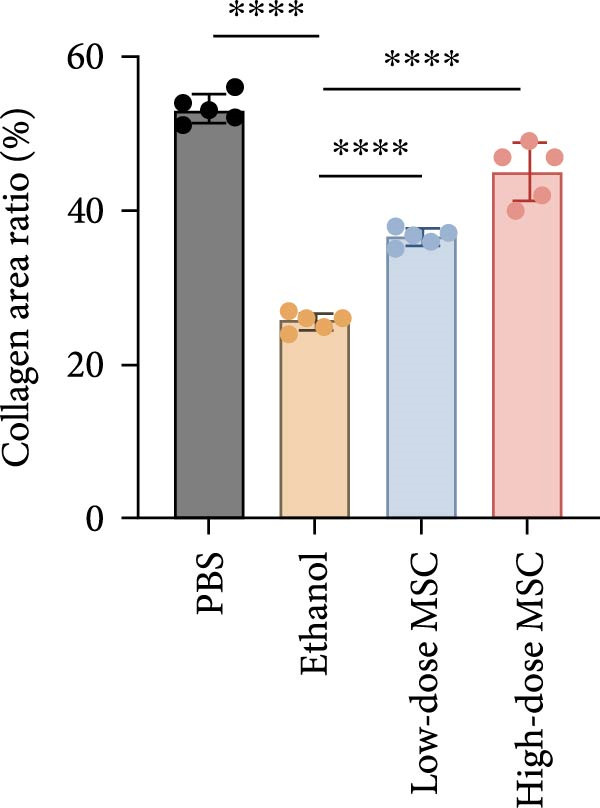
(D)

**Figure:**
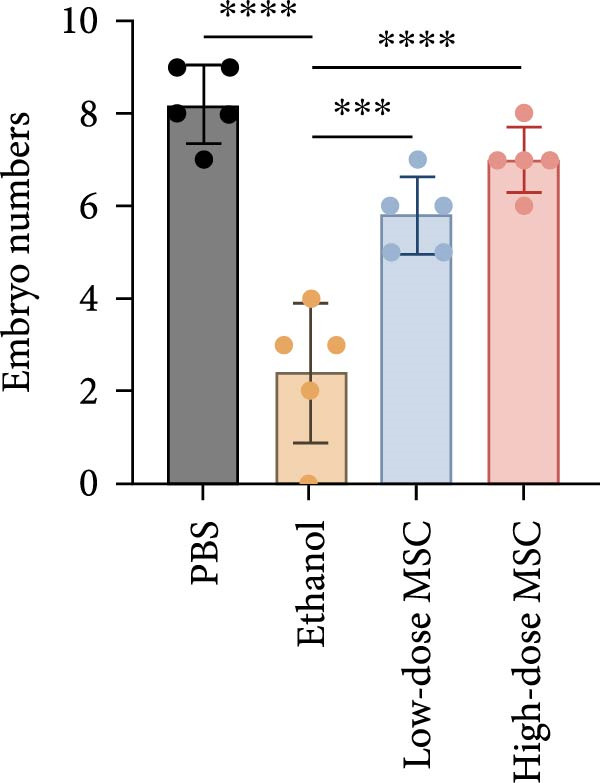
(E)

**Figure:**
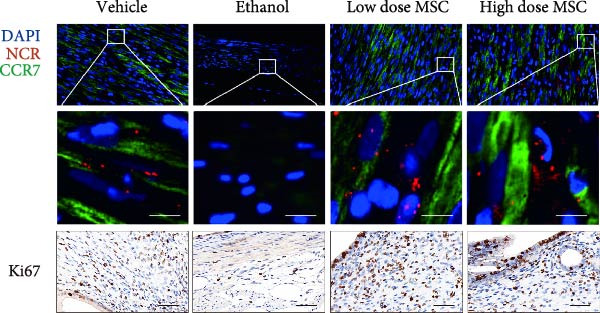
(F)

**Figure:**
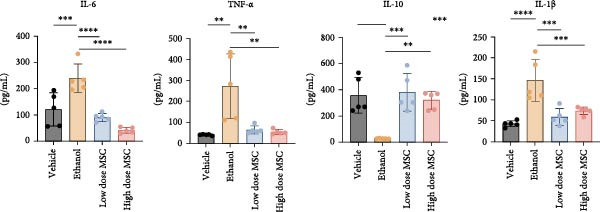
(G)

### 3.5. CCR7 Upregulation Mediates MSCs‐Induced ERK/JNK Pathway Activation in NK92 Cells

Figure 5A illustrates the coculture system of MSCs and NK92 cells, providing a visual framework for the in vitro experiments presented in panels B, C, and D. Western blot analysis demonstrated density‐dependent CCR7 upregulation in NK92 cells following MSCs coculture, with p‐JNK and p‐ERK expression levels correlating with CCR7 expression and peaking at 1 × 10^4^ and 2 × 10^4^ MSCs/mL. No significant changes were observed in p‐P38, JNK, ERK, or P38 expression (Figure 5B). To validate pathway‐specific causal relationships, we examined the effects of SP600125 (SP, a p‐JNK inhibitor) and PD98059 (PD, a p‐ERK inhibitor) in 2 × 10^4^ MSCs/mL cocultures. As shown in Figure 5C, SP treatment significantly reduced p‐JNK protein expression in NK cells cocultured with 2 × 10^4^ MSCs/mL, while PD treatment markedly decreased p‐ERK protein expression, confirming that ERK/JNK (rather than p38) mediates MSC‐induced signaling. CCR7 knockdown using siRNA reduced ERK/JNK expression levels, an effect further validated by MSCs intervention. These results collectively demonstrate that MSCs‐mediated MAPK pathway activation occurs through CCR7 upregulation (Figure 5D).

Figure 5CCR7 upregulation mediates MSCs‐induced ERK/JNK pathway activation in NK92 cells (A) Schematic of transwell coculture system with MSCs (upper chamber) and NK92 cells (lower chamber) separated by a semipermeable membrane. (B) Dose‐responsive modulation of CCR7 and ERK/JNK signaling components in NK92 cells following MSC coculture. (C) p‐JNK and p‐ERK expression in NK cells. (D) CCR7 and ERK/JNK signal components in NK92 cells of two experimental groups: NC (untreated control) and MSCs. Each group was further divided into two subgroups: NC and sir‐CCR7 (treated with siRNA targeting CCR7).

**Figure:**
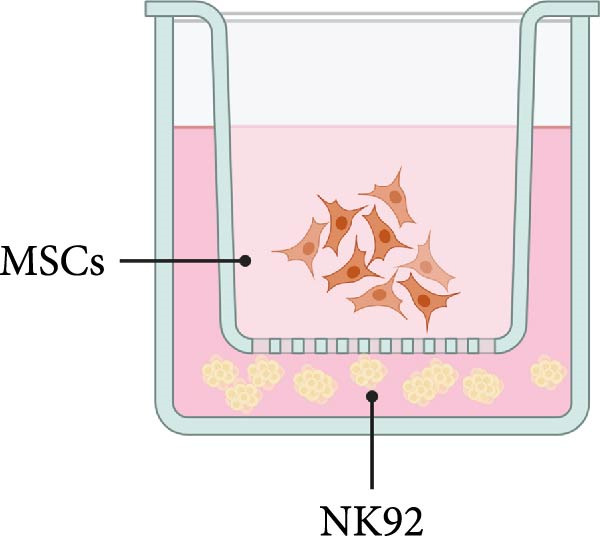
(A)

**Figure:**
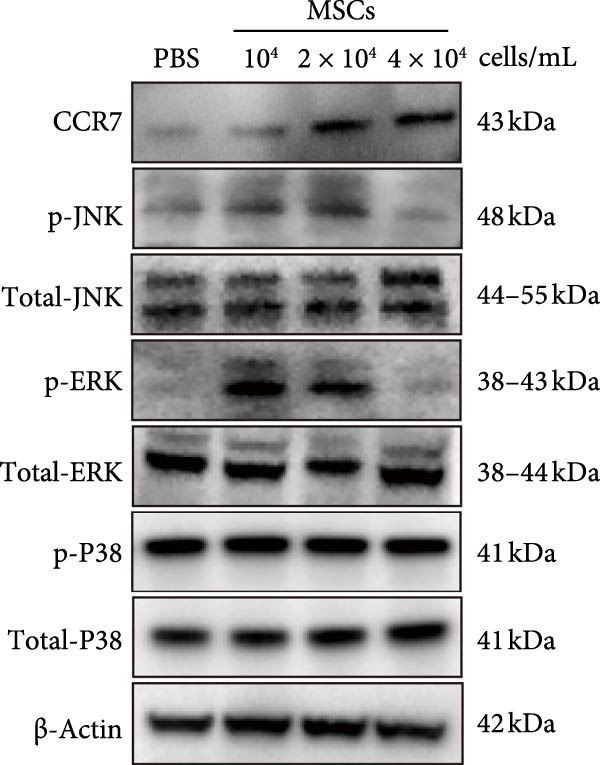
(B)

**Figure:**
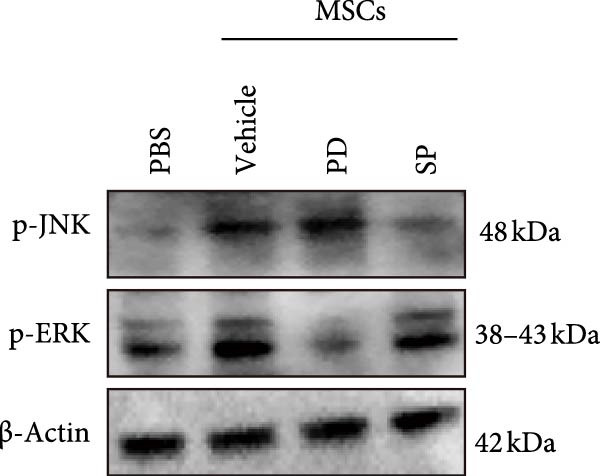
(C)

**Figure:**
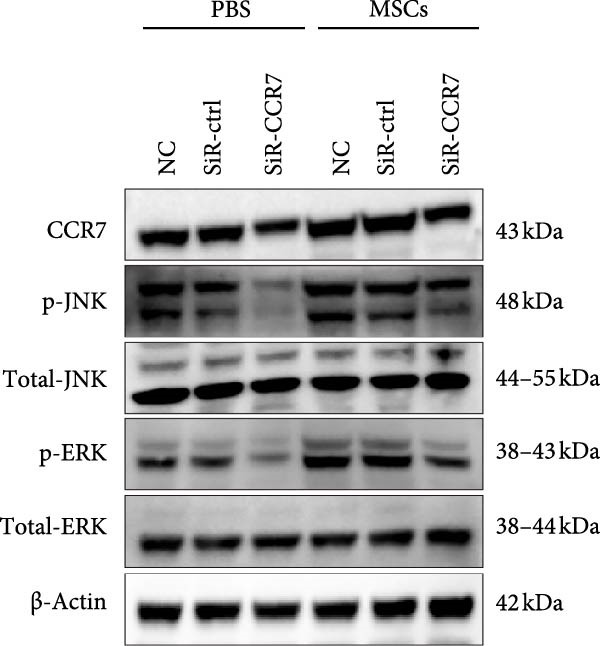
(D)

### 3.6. MSCs Therapy Enhances Endometrial Proliferation Through CCR7/ERK/JNK Signaling

In a rat model of ethanol‐induced endometrial injury, different doses of MSCs (10^5^, 2 × 10^5^, and 4 × 10^5^ cells/rat) were administered to evaluate therapeutic effects. Results showed that the positive expression areas of p‐JNK and p‐ERK gradually expanded with increasing MSC doses, and the percentage of Ki67‐positive cells also increased in a dose‐dependent manner (Figure 6A‐D). These findings indicate that MSCs promote endometrial cell proliferation through CCR7 upregulation and JNK/ERK pathway activation, exhibiting a dose‐dependent therapeutic effect. To further investigate the role of CCR7, rats with ethanol‐induced injury were treated with MSCs at the 10^5^ cells/rat dose, and a CCR7 antagonist was administered concurrently. Relative to vehicle controls, the antagonist significantly suppressed both p‐JNK and p‐ERK phosphorylation in engrafted MSCs, suggesting that CCR7 orchestrates MAPK signaling pathway activation during MSC‐mediated endometrial tissue repair (Figure 6E). Pharmacological inhibition experiments further confirmed pathway specificity: PD (20 mg/kg, intravenous as p‐ERK inhibitor) markedly reduced p‐ERK levels, while SP (1 mg/kg, intraperitoneal as p‐JNK inhibitor) significantly decreased p‐JNK expression in MSCs‐treated rats (Figure 6F). Immunohistochemical staining for Ki67 revealed that MSC treatment significantly increased the number of Ki67‐positive cells compared with the PBS control group, indicating enhanced endometrial cell proliferation. Notably, this MSC‐induced proliferative effect was blocked by coadministration of SP600125 or PD98059, suggesting dependence on JNK/ERK pathway activation. Quantitative analysis further confirmed that the percentage of Ki67‐positive cells in the MSCs group was remarkably higher than in the PBS group, whereas the MSCs + PD and MSCs + SP groups exhibited significantly lower percentages relative to the MSCs alone group (Figure 6G‐H). Collectively, these results demonstrate that MSCs enhance endometrial proliferation through CCR7‐mediated activation of the JNK/ERK signaling pathway, with a dose‐dependent therapeutic effect in endometrial injury repair.

Figure 6MSCs therapy enhances endometrial proliferation through CCR7/ERK/JNK signaling. (A) Immunostaining of p‐JNK, p‐ERK, and Ki67 in ethanol‐induced rats treated with PBS or MSCs (1 × 10^5^, 2 × 10^5^, 4 × 10^5^ cells/rat). Scale bar:50 μm. (B–D) Quantitative analysis of p‐JNK, p‐ERK, and Ki67 positive rates (*n* = 4/group, “ns” stands for *p*＞ 0.05,  ^∗^ *p* < 0.05,  ^∗∗^ *p* < 0.01,  ^∗∗∗^ *p* < 0.001,  ^∗∗∗∗^ *p* < 0.0001 vs. ethanol group). (E) Western blot analysis of CCR7, p‐JNK, and p‐ERK expression in PBS control and MSCs‐treated groups with vehicle or CCR7 antagonist in vivo. (F) p‐JNK and p‐ERK levels in PBS control and MSCs‐treated groups with PD or SP. (G) Representative Ki67 immunohistochemistry in PBS, MSCs, MSCs + PD, and MSCs + SP groups. Scale bar:50 μm. (H) Quantification of Ki67^+^ cells (*n* = 4/group,  ^∗∗∗∗^ *p* < 0.0001 vs. MSCs group).

**Figure:**
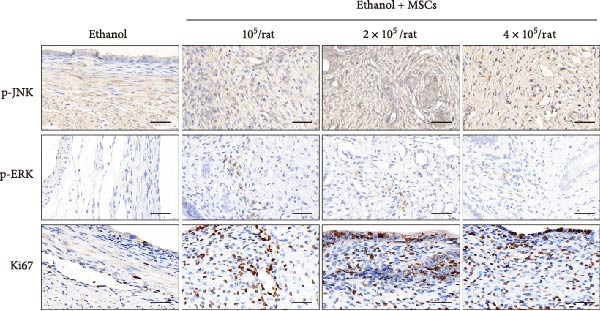
(A)

**Figure:**
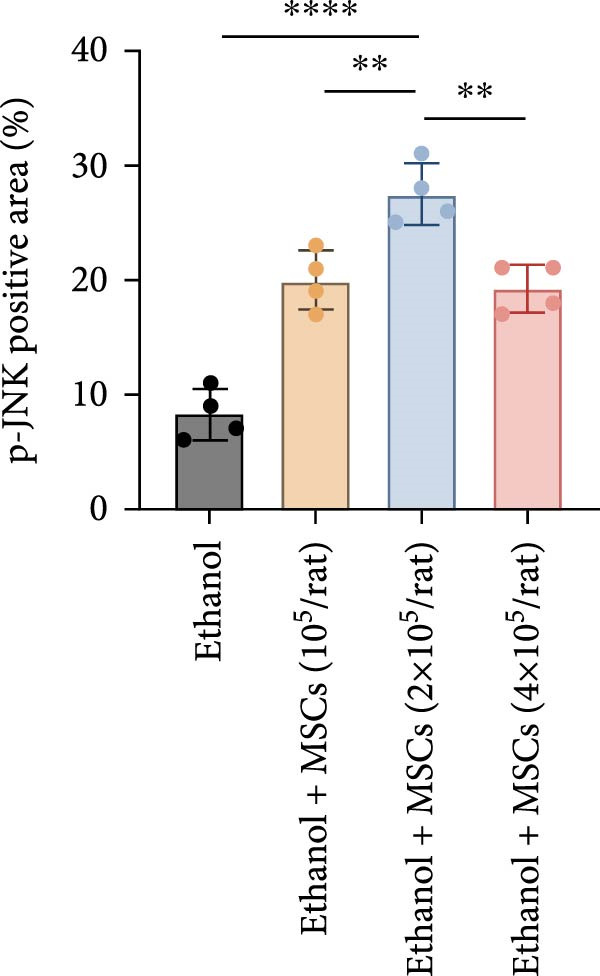
(B)

**Figure:**
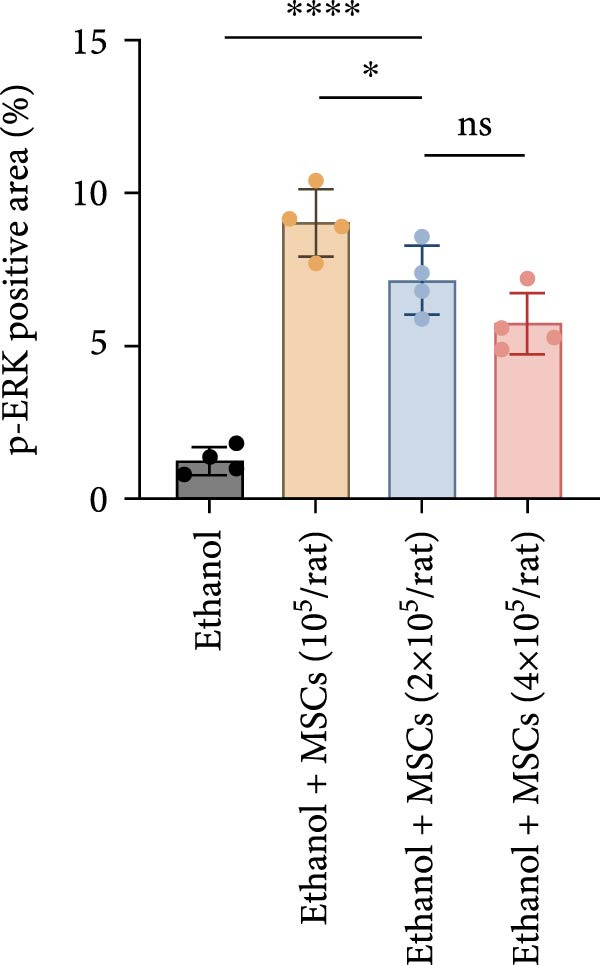
(C)

**Figure:**
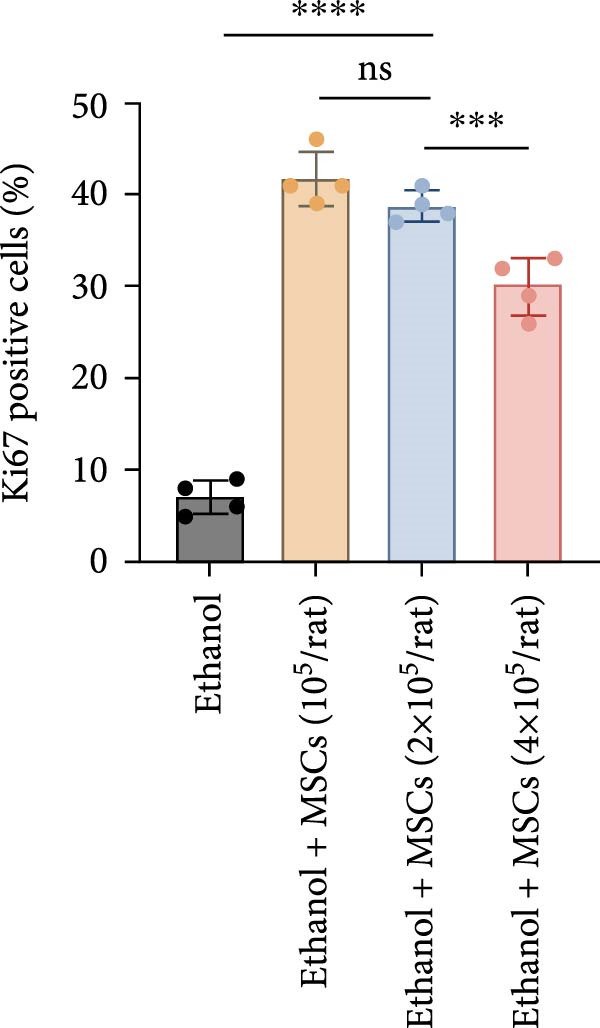
(D)

**Figure:**
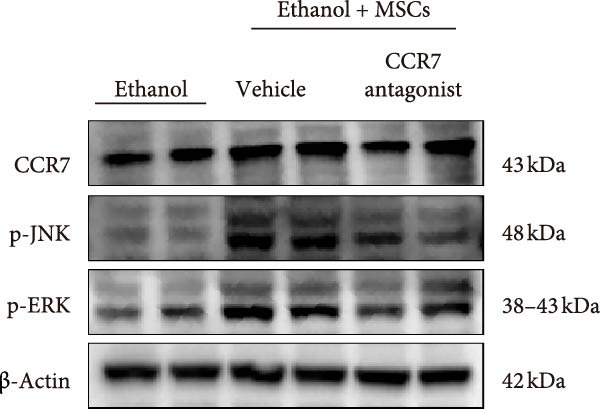
(E)

**Figure:**
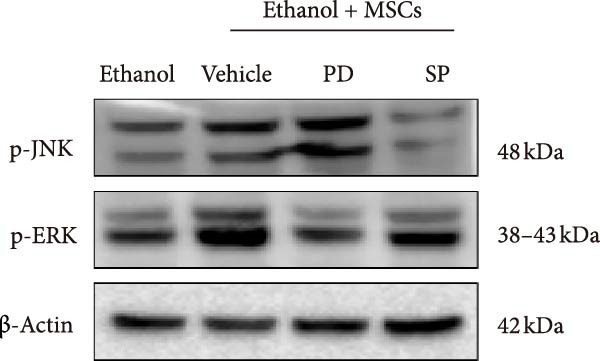
(F)

**Figure:**
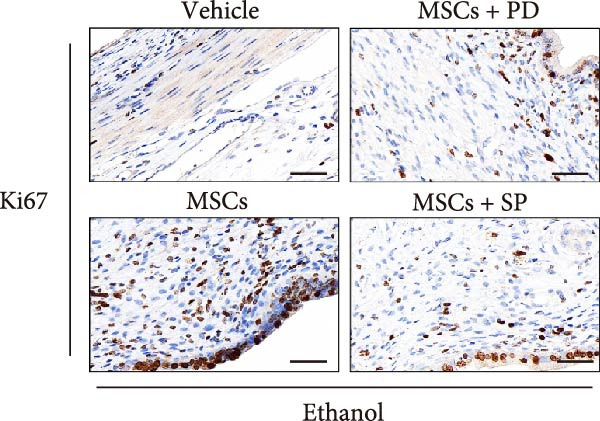
(G)

**Figure:**
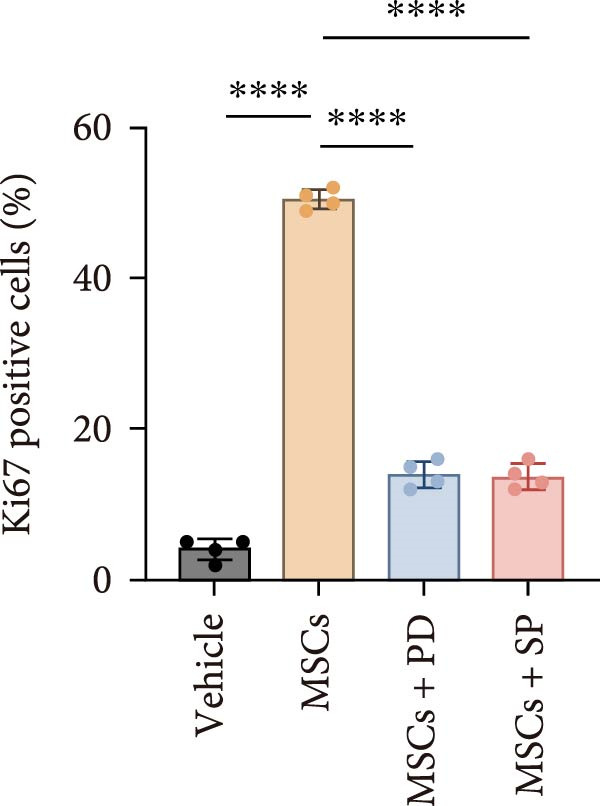
(H)

## 4. Discussion

In this study, we demonstrate the therapeutic mechanisms by which UC‐MSCs prevent RPL through bidirectional interactions with uNK cells and endometrial stromal repair. Our findings reveal that UC‐MSCs exert their therapeutic effects via two complementary pathways:CCR7/ERK/JNK‐mediated uNK cell modulation and endometrial regeneration. Unlike previous studies highlighting CCR7’s role in dendritic cell migration or broad chemokine suppression in GVHD [18, 19], our article uniquely identifies CCR7 as a key mediator of UC‐MSCs’ tissue‐repairing capacity through Extl1‐independent stromal‐immune crosstalk. By elucidating these dual therapeutic mechanisms, we propose a targeted UC‐MSCs‐based strategy distinct from conventional anti‐inflammatory or DC‐centric approaches.

CCR7, traditionally known for its role in lymphocyte homing to lymphoid organs, has recently been implicated in uNK cell migration during early gestation [20]. Our RNA‐Seq analysis and immunofluorescence data demonstrate that MSCs transplantation significantly upregulates CCR7 expression in uNK cells, which correlates with enhanced migratory capacity toward inflammatory chemokines such as CCL19 and CCL21. This suggests that MSCs promote uNK cell recruitment to the endometrium through CCR7‐mediated chemotaxis. The ERK/JNK signaling pathways are known regulators of NK cell chemotaxis and cytotoxic granule polarization [21, 22]. Our results show that MSCs induction of NK cells leads to a transient but potent phosphorylation of ERK1/2 and JNK, indicating their activation. Using specific inhibitors, we further validated that these signaling pathways are critical for MSCs‐mediated regulation of NK cell function. Moreover, immunohistochemical analysis of endometrial sections revealed that MSCs treatment enhances ERK/JNK signaling activation in a dose‐dependent manner, suggesting its involvement in endometrial stromal repair. Our findings provide a mechanistic basis for MSCs based therapies in RPL. By restoring endometrial stromal homeostasis and reprograming uNK cell dynamics, MSCs offer a promising approach to prevent pregnancy loss in patients with unexplained RPL. The identified CCR7‐ERK/JNK axis not only enhances our understanding of the complex interplay between immune cells and stromal compartments during pregnancy but also suggests potential therapeutic targets for improving pregnancy outcomes.

While our study demonstrates the therapeutic potential of MSCs in preventing RPL through CCR7‐mediated endometrial repair and uNK cell immunomodulation, we acknowledge that the immunomodulatory effects of MSCs on NK cells are well‐documented in existing literature. Previous studies have shown MSCs regulate NK cell function through multiple established mechanisms, including secretion of IDO, PGE2, and soluble HLA‐G5, as well as induction of anti‐inflammatory purinergic signaling in activated NK cells [23, 24]. Our study builds upon these findings by uniquely demonstrating the synergistic effects of CCR7‐mediated endometrial repair and uNK cell immunomodulation in improving pregnancy outcomes, which represents a novel therapeutic strategy for RPL. The ethanol‐induced endometrial injury model, while useful for studying chemical injury, has several limitations in accurately modeling RPL: (1) it primarily induces acute inflammation rather than replicating the complex immune‐endocrine interactions of RPL; (2) most studies using this model focus on endometrial structure rather than pregnancy maintenance, which is central to RPL pathology; and (3) species‐specific responses may limit translational relevance to human RPL pathophysiology [25, 26]. Additionally, the rat model may not fully capture the complexity of human pregnancy, necessitating further validation in larger animal models or clinical trials. Future research should focus on identifying the soluble factors released by MSCs that mediate these effects and exploring their potential as therapeutic agents.

In conclusion, our study demonstrates that MSCs exert therapeutic effects through the novel CCR7‐ERK/JNK axis, orchestrating endometrial stromal repair and modulating uNK cell dynamics to sustain pregnancy. These findings highlight MSCs as a promising therapeutic strategy for RPL, offering a dual mechanism of action that integrates tissue regeneration and immune regulation. This article provides a rationale for targeted MSCs‐based therapies in clinical management of idiopathic RPL.

**Nomenclature**
CCR7: C‐C chemokine receptor type 7
ERK: Extracellular signal‐regulated kinase
JNK: C‐Jun N‐terminal kinase
MSC: Mesenchymal stem cell
RPL: Recurrent pregnancy loss
uNK: Uterine natural killer
IL‐6: Interleukin‐6
TNF‐α: Tumor necrosis factor‐alpha
IL‐1β: Interleukin‐1 Beta
IL‐10: Interleukin‐10
ELISA: Enzyme‐linked immunosorbent assay
GO: Gene ontology
KEGG: Kyoto Encyclopedia of genes and genomes
DEGs: Differentially expressed genes
MHC: Major histocompatibility complex
FBS: Fetal bovine serum
PFA: Paraformaldehyde
SEM: Standard error of the mean
IHC: Immunohistochemistry
FACS: Fluorescence‐activated cell sorting
RPL: Recurrent pregnancy loss
MAPK: Mitogen‐activated protein kinase
p‐ERK: Phosphorylated extracellular signal‐regulated kinase
p‐JNK: Phosphorylated c‐Jun N‐terminal kinase.

## Conflicts of Interest

The authors declare no conflicts of interest.

## Author Contributions

Yunguo Lei, Juanmei Gao, and Ning Zhang contributed equally to this work.

## Funding

This study was funded by the Traditional Chinese Medicine Science and Technology Plan Project of Zhejiang Province (Grant 2023ZL566), the Key Project of Hangzhou Science and Technology (Grant ZD20220060), and the National Key Research and Development Program of China (Grant 2021YFA1100500).

## Data Availability

The data that support the findings of this study are available upon request from the corresponding author.
